# Astragalus membranaceus and Punica granatum alleviate infertility and kidney dysfunction induced by aging in male rats

**DOI:** 10.3906/biy-2001-5

**Published:** 2020-08-19

**Authors:** Ameera S. ALSHINNAWY, Wael M. EL-SAYED, AlShaimaa M. TAHA, Ahmed A. SAYED, Ahmed M. SALEM

**Affiliations:** 1 Department of Biochemistry, Faculty of Science, Ain Shams University, Cairo Egypt; 2 Department of Zoology, Faculty of Science, Ain Shams University, Cairo Egypt; 3 Children’s Cancer Hospital, Cairo Egypt

**Keywords:** Infertility, FSH, LH, testosterone, sperm analysis, kidney dysfunction

## Abstract

By aging, male fertility and kidney function decline. Therefore, the investigation of health span-extending agents becomes more urgent to overcome aging-induced infertility and kidney dysfunction. The current research was undertaken to investigate the antiaging efficacy of *Astragalus membranaceus* telomerase activator-65 (Ta-65) and pomegranate supplements. Forty male Wistar rats were divided into young rats, aged rats, aged rats treated with Ta-65 (500mg/kg/day), and aged rats treated with pomegranate (250mg/kg/day). Testosterone, FSH, LH, and kidney functions were measured in serum. Sperm analysis as well as testicular histological examination was performed. Aging caused an imbalance in male sex hormones resulting in sperm abnormality and reductions in the sperm count and motility. Elevations in serum creatinine, uric acid, sodium, and potassium were reported in aged rats. Treatment with Ta-65 or pomegranate effectively ameliorated all the deteriorations induced by normal aging in male fertility and renal function. Ta-65 and pomegranate possessed strong antiaging activity by alleviating aging-induced male infertility through reestablishing the hormonal balance and testis architecture. They also alleviated the kidney dysfunction. On comparing Ta-65 with pomegranate, the improvement in FSH, LH, and sperm abnormalities caused by Ta-65 was much better than that caused by pomegranate.

## 1. Introduction

Infertility affects up to 20% of couples globally (Pan et al., 2018). The risk of poor reproductive insufficiency in males increases at >35 years old due to age-related changes in aged males including high incidence of systemic diseases and infections, vascular insufficiency, reduced levels of sex hormones, mutations, disorders in the architecture of testes, and oxidative stress (Azenabor et al., 2015; Ilacqua et al., 2018). In men over 35 years old, negative changes in sperm quality are reported and become more pronounced with age over 40 years (Rosiak-Gill et al., 2019). By aging, deterioration in semen quality is observed in the ejaculate volume, sperm count, motility, vitality and morphology (Harris et al., 2011). 

Furthermore, aging is associated with loss of kidney function marked with progressive reductions in renal blood flow and glomerular filtration rate (GFR). The decline in GFR is attributed to reductions in glomerular capillary flow rate, glomerular capillary ultrafiltration coefficient, and afferent arteriolar resistance (Weinstein and Anderson, 2010). These changes lead to loss of renal mass; stiffness of afferent arterioles, sclerosis of glomeruli, and tubulointerstitial fibrosis (Yoon and Choi, 2014). Aging is associated with altered activity and responsiveness to vasoactive stimuli; responses to vasoconstrictor stimuli are enhanced, while vasodilatory responses are impaired. These changes may predispose the older kidney to acute and progressive chronic kidney injury (Weinstein and Anderson, 2010).

Therefore, targeting the pathological changes associated with male infertility and kidney dysfunctions could reverse these aging-induced perturbations. *Astragalus membranaceus* is a Chinese medicinal herb used to treat various diseases and is marketed as life-prolonging extracts for human use in China (Liu et al., 2017). The major components of *Astragalus membranaceus* are polysaccharides, flavonoids, and saponins (Auyeung et al., 2016). The components of *Astragalus membranaceus *extract can increase telomerase activity, and has antiinflammatory, antioxidant, anticancer, immunoregulatory, antihyperglycemic, hypolipidemic, expectorant, hepatoprotective, and diuretic effects (Liu et al., 2017). TA-65, the extract of dried root of *Astragalus membranaceus*, is known with a significant age-reversal effect in the immune system (Liu et al., 2017).

Peels of pomegranate (*Punica granatum* L.) contain high content of polyphenols such as condensed tannins and proanthocyanidins, anthocyanins, and flavonoids. Pomegranate peels havecytotoxic (Kulkarni et al., 2007), hepatoprotective (Chidambara et al., 2002), and hypoglycemic activities (Hontecillas et al., 2009). 

There are no available data about the effects of Ta-65 and pomegranate on male infertility and kidney dysfunction produced by normal aging. Rat animal model (normal aging) was chosen for the present study to evaluate the efficacy of Ta-65 and pomegranate extracts on male gonad function, semen quality, testes histology, and kidney function tests. 

## 2. Materials and methods

### 2.1. Preparation of Ta-65 and pomegranate supplements

The powders of Ta-65 (RD Health Ingredients Co., Ltd., Shaanxi, China, CAS: 78574-94-4) extracted from roots of *Astragalus membranaceus* as well as pomegranate (Wuhan HengHeDa Pharm Co., Ltd., Shanghai, China, CAS: 476-66-4) extracted from peels of *Punica granatum* were freshly dissolved in sterile saline solution. 

### 2.2. Animals

A total of 30 adult male Wistar rats (3 months) weighing 100 ± 20 g and then ten newborn male Wistar rats (3 weeks) weighing 15 ± 5 g were provided from the animal house of the Egyptian Company for Vaccines and Sera (VACSERA, Giza, Egypt) in an air-conditioned car. The adult rats were left for seven months without any treatment until reaching 10-month-old (aged rats) at 22 ± 2°C and 40%–60% humidity with natural light and dark cycles (12/12 h). Within this period, the newborn rats were brought and left for one month until reaching 2 months old at the same conditions of temperature and humidity. Five rats were housed in steel mesh cages with water and a commercial pellet diet ad libitum. The experiment was performed in the animal house at the Department of Zoology, Faculty of Science, Ain Shams University. Research Ethics Committee (REC) for animal research at National Hepatology and Tropical Medicine Research Institute (NHTMRI) has approved the research protocol (Serial#18-2018).

### 2.3. Study design

The rats were randomly divided into four groups as follows; Group I (young control); 10 young rats (2-month-old) were treated orally with saline for two consecutive months until reaching 4 months old. Group II (aged control); 10 old adult rats (10-month-old) were administered orally with saline for two consecutive months until reaching one years old. Group III (aged+Ta-65); 10 old adult rats (10-month-old) were treated orally for two consecutive months with Ta-65 supplement (500 mg/kg/day, Murbach et al., 2019) using oral gavage. Group IV (aged + pomegranate); 10 old adult rats (10-month-old) were treated orally with pomegranate extract at a dose of 250 mg/kg/day (Ahmed et al., 2014) using oral gavage for two consecutive months.

### 2.4. Blood sampling and collection of body organs

At the end of the experimental period, the animals were fasted for 8 h, weighed, anesthetized then sacrificed by cervical dislocation and blood was immediately collected via heart puncture. For separation of serum samples, blood samples were incubated for 30 min at 37 °C, centrifuged at 1500 × g at 4 ºC for 15 min. Serum was separated, aliquoted, and stored at –80 °C until analyses. Kidneys and testes were excised, rinsed thoroughly in isotonic sterile saline containing heparin, blotted dry with a filter paper and weighed. The relative weight ratios of kidneys and testes in all studied groups were calculated. One testis from each rat was fixed in 10% formalin solution for at least 3 days at 4 °C for histopathological examination. 

### 2.5. Histopathological examination

Small specimens of testis from the different groups were fixed in 10% formalin solution for 2 days at 4 ˚C. The fixed specimens were washed with tap water then serial dilutions of alcohols (methyl, ethyl and absolute ethyl) were used for dehydration. Specimens were cleared in xylene and embedded in paraffin at 56 ˚C in hot air oven for 24 h. Paraffin bees wax tissue blocks were prepared for sectioning at 4 μm by slidge microtome. The obtained tissue sections were collected on glass slides, deparaffinized and stained by hematoxylin and eosin stains (Drury, 1983) for histopathological examination under the electric light microscope (Olympus Corporation, Tokyo, Japan) at a magnification power × 200, × 400, and × 1000.

### 2.6. Sperm analysis

The count, motility percentage, and morphology of sperms were performed as described elsewhere (Oliveira et al., 2014). 

#### 2.6.1. Sperm count

The cauda epididymis and upper portions of the vas deferens of rats were cleaned from any adherent tissue, and then placed in a plate that contained normal saline for washing out the blood. The cauda epididymis was cut longitudinally into pieces to release sperm into a Petri dish containing 2 ml of isotonic saline at 37 °C. The sperm suspensions were incubated for 10 minutes at 37 °C to completely allow the spermatozoa to flow out from cauda epididymis into the saline. A volume of 100 µL of sperm suspensions was added to 900 µL isotonic buffer. A volume of 10 µL of the diluted sperm suspension was placed under cover slip of hemocytometer to be counted.

Calculation:

Sperms count/mL = Total no. of sperms in 5 squares × 50 × 1000 × Dilution Factor

#### 2.6.2. Sperm motility 

A volume of 10 µL of the diluted sperm suspension was loaded under the cover slip of hemocytometer.

Calculation:

The numbers of progressive motile, nonprogressive motile and immotile sperms were counted and then the percentage was calculated as follows:

% of progressive motile sperms = 

(Number of progressive motile sperms/total number of sperms in fixed square) × 100

% of nonprogressive motile sperms = 

(Number of nonprogressive motile sperms/total number of sperms in fixed square ) × 100

% of immotile sperms = 

(Number of immotile sperms/total number of sperms in fixed square) × 100

#### 2.6.3. Sperm morphology 

Sperm morphology is monitored by smearing the sperm sample on a clean, grease-free microscope slide followed by examination under a light microscope under the oil immersion objective lens. A volume of 10 µL of sperm suspension was smeared on a microscope slide. Smears were air-dried then fixed in absolute methanol. The slides were stained with 1% aqueous Eosin-Y solution for an hour, washed with distilled water, passed through neutral resin, and mounted with coverslips.

Calculation:

A 200 µL sample was counted using the oil immersion objective lens, and classified as follows; normal, big head, hookless head, amorphous head, compacted head, tailless head, abaxial tail attachment, U-shaped tail, highly folded tail, coiled tail, and headless tail. Sperm abnormalities were recorded as percentages of the total number of counted spermatozoa.

### 2.7. Male sex hormones profile

The levels of follicle stimulating hormone (FSH), luteinizing hormone (LH), and testosterone were measured in serum using sandwich ELISA research kits purchased from MyBioSource, Inc. (San Diego, CA, USA) following the instructions of the manufacturer.

### 2.8. Kidney function tests

Serum levels of creatinine, uric acid, sodium, and potassium were estimated using commercial colorimetric assay kits (Spectrum Diagnostics, Obour City, Egypt) following the instructions of the manufacturer. 

### 2.9. Statistical analysis

Statistical analysis was performed using the Statistical Package for Social Science version 20.0 for Windows (SPSS 20.0, IBM Corp., Armonk, NY, USA). Distribution of data was tested using the Kolmogorov–Smirnov test. Individual data in experimental groups were analyzed using one-way Analysis of Variance (ANOVA). To compare the difference between the groups, post hoc testing was performed by Tukey’s test for multiple comparisons between the different treated groups and their respective controls. P values were considered significant at P < 0.05.

## 3. Results

### 3.1. Effect on body and organ weights

**Figure 1:**
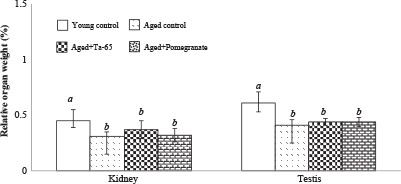
Toxicological effects of Ta-65 and pomegranate supplements on the relative weight ratios of kidney and testes. Data are expressed as Mean ± SD, n = 8. Different characters denote significance between groups. The mean difference is significant at P < 0.05.

Normal aging significantly declined the relative ratios of kidney (31.38%) and testis (32.39%), compared to the young rats. The reductions in the relative kidney and testis ratios were not attenuated after the daily oral administrations of either Ta-65 or pomegranate (Figure 1).

### 3.2. Effect on male fertility

**Figure 2:**
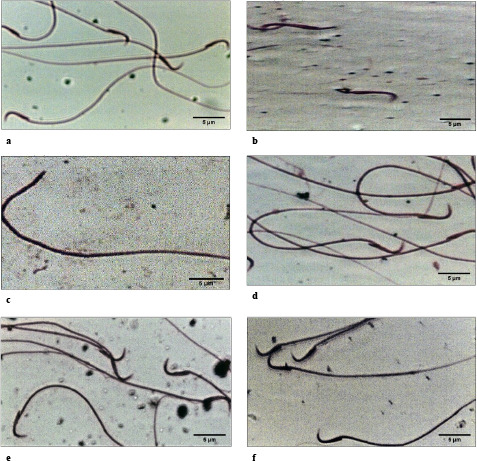
Photomicrographs of epididymal sperm smears stained with Eosin-Y (H&E, 1000x). a: young control group, b–d: aged control group; e: Aged+Ta-65 group; f: Aged+pomegranate group. The forms of morphological abnormalities were tailless head, headless tail, and U-shaped tail.

Results represented in Table 1 and Figure 2 demonstrate that aging decreased the male fertility by significant reductions in sperms count (51.88%), sperms motility percentage (82.19%), in association with significant elevations in the percentages of sperms abnormality (269.07%) and immotility (130.77%), compared to the young rats. Aging induced sperm morphological abnormalities in the form of tailless, headless, or sperms with U-shaped tail. Treatment of aged rats with Ta-65 attenuated male fertility by increasing both sperm count (93.08%) and motility percentage (438.46%) as well as decreasing the percentage of morphology abnormalities in morphology (71.23%), compared to aged rats. Similarly, oral administration of pomegranate improved the percentage of both sperms abnormalities and motility, compared with aged control rats, but this improvement in sperm abnormalities remained significantly different from that of young rats (52.58%). On comparing Ta-65 group with pomegranate group, there were no significant changes in the sperms count and sperms motility percentage. However, the percentage of sperms abnormalities was found to be significantly higher than the young control rats after pomegranate treatment (43.69%).

**Table 1:** Effect of antiaging supplements on the sperm analysis and male sex hormones profile.

	Young control	Aged control	Aged+Ta-65	Aged+pomegranate
Sperm count (× 10^6^/ml)	113.2 ± 8.02^a^	54.47 ± 6.77^b^	105.17 ± 13.72^a^	100.20 ± 14.14^a^
Sperm abnormality (%)	8.08 ± 2.60^a^	29.83 ± 3.60^b^	8.58 ± 0.38^a^	12.33 ± 0.41^c^
Progressive motile sperms (%)	48.67 ± 1.86^a^	8.67 ± 3.27^b^	46.67 ± 1.97^a^	45.50 ± 3.14^a^
Nonprogressive motile sperms (%)	16.67 ± 1.51^a^	11.33 ± 2.07^b^	15.33 ± 1.97^a^	17.00 ± 1.55^a^
Immotile sperms (%)	34.67 ± 2.94^a^	80.00 ± 1.90^b^	38.00 ± 2.76^a^	37.50 ± 2.74^a^
FSH (pg/mL)	5.24 ± 0.45^a^	1.88 ± 0.28^b^	3.46 ± 0.15^c^	2.82 ± 0.22^d^
LH (pg/mL)	7.16 ± 0.55^a^	2.00 ± 0.10^b^	4.29 ± 0.53^c^	3.26 ± 0.29^d^
Testosterone (pg/mL)	4.40 ± 0.20^a^	1.81 ± 0.08^b^	3.83 ± 0.28^c^	3.46 ± 0.56^c^

-Results are expressed as Mean ± SD, n = 8. -Different characters denote significance between groups. -The mean difference is significant at P < 0.05.

Significant reductions in the serum levels of testosterone, FSH, and LH of aged rats (58.74%, 64.12, and 72.04, respectively) were reported compared to the young rats (Table 1). Administration of Ta-65 to aged rats improved serum levels of testosterone, FSH, and LH compared to the aged group, but the levels were still significantly lower than the normal young levels by 13.13, 33.97, and 40.02%, respectively. Treatment of aged rats with pomegranate also improved the serum levels of testosterone, FSH, and LH compared with the aged rats, but the levels were still significantly lower than the normal young level by 21.5, 46.18, and 54.41%, respectively. Moreover, there were significant reductions in FSH (18.32%) and LH (23.99%) in serum of aged rats treated with pomegranate compared to aged rats treated with Ta-65 but no significant change in serum testosterone level was reported between the two groups.

### 3.3. Effect on the architecture of testes

**Figure 3:**
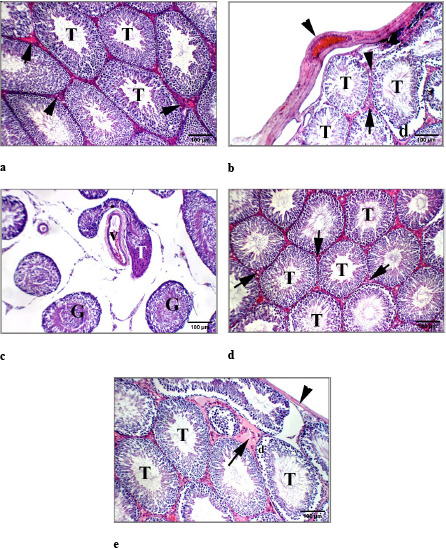
Photomicrographs of testes sections stained with hematoxylin and eosin staining (H&E, 200x). Young control (a), Aged control (b and c), Aged+Ta-65 (d), Aged+pomegranate (e). The testis of young rats showed average-sized tubules with full spermatogenesis (T) with an average interstitium (arrow). The aged rats had testis with mildly thickened tunica albuginea (arrow head) and average-sized tubules (T) with detached germinal lining (d) and average interstitium (arrow) (b). In addition, a widely-separated distorted partially sclerotic tubule (T) could be seen, other tubules showed mildly desquamated germinal cells (G) with dilated interstitial blood vessel (V) (c). Oral administration of Ta-65 to aged rats showed testis with showing average tubules with average germinal lining (T) and average interstitium (arrow). Testis of rats treated with pomegranate revealed average tunica albuginea (arrow head), average-sized tubules with detached germinal lining (d) and mild interstitial edema (arrow).

The Figure 3 and Table 2 demonstrate the histological examination of the H&E-stained cross sections of testis. Young rats showed rounded or oval seminiferous tubules with normal histological structures (Figure 3a). The tubules were lined with a complex stratified epithelium with different stages of spermatogenesis (spermatogonia, primary spermatocytes, secondary spermatocytes, spermatids, and spermatozoa). In addition, the tubules were separated by an interstitial tissue and the lumina of tubules were filled with spermatozoa. Groups of interstitial cells of Leydig were seen between the seminiferous tubules. On the other hand, cross section of testis of aged rats illustrated a disturbance in the normal architecture with structural changes. Some of the seminiferous tubules were widely separated from each other (Figure 3b). In addition, the basement membrane was focally separated from the overlying germinal epithelium. The interstitial spaces revealed deposition of homogeneous acidophilic material (Figure 3c). Some spermatogenic cells appeared disorganized with wide intercellular spaces and others with deeply stained nuclei were exfoliated into the lumen of the tubules. Partially severe sclerotic tubule with no spermatogenesis and desquamated germinal cells also appeared. Oral administration of Ta-65 to aged rats caused a partial preservation of the normal structure of seminiferous tubules, and interstitial cells of Leydig. Some spermatogenic cells showed mild cytoplasmic vacuolation (Figure 3d). The lumina of tubules were full with spermatozoa. Oral administration of pomegranate to aged rats resulted in an improvement in the tubular basal compartment with the presence of normal spermatogonia, a decrease in extracellular matrix in interstitial space, and appearance of seminiferous tubules with normal spermatids in the luminal compartment (Figure 3e).

**Table 2:** Scoring of different histological abnormalities in the testes of different groups.

Abnormalities	Young control	Aged control	Aged+Ta-65	Aged+pomegranate
Thickened capsule	0	+	0	0
Distorted/sclerotic T.	0	++	+	+
Atrophied germinal lining	0	++	+	+
Disorganized Spermatogenesis	0	+	0	0
Separated interstitium T.	0	++	+	+
Desquamated cells	0	++	+	++
Dilated/congested B.V.	+	+	+	0

-T= Tubules, B.V. = Blood vessels. -0 means normal, + means mild, ++ means moderate.

### 3.4. Effect on kidney functions

Data illustrated in Table 3 reveal significant elevations in the levels of creatinine (51.43%) and uric acid (52.69%) in serum of aged rats, compared to the young rats, while serum levels of sodium and potassium were significantly decreased (19.81% and 48.07%, respectively). In aged rats administered with both Ta-65 and pomegranate, serum levels of creatinine, uric acid, sodium, and potassium were sustained to the normal levels seen in young rats. There were nonsignificant differences in serum levels of creatinine, uric acid, sodium, and potassium between aged + Ta-65, and aged + pomegranate groups.

**Table 3:** Effect of Ta-65 and pomegranate supplements on serum kidney function tests.

	Young control	Aged control	Aged+Ta-65	Aged+pomegranate
Creatinine (mg%)	0.45 ± 0.07^a^	0.71 ± 0.12^b^	0.45 ± 0.08^a^	0.46 ± 0.07a
Uric acid (mg%)	13.36 ± 1.50^a^	20.40 ± 2.47^b^	12.84 ± 0.83^a^	14.51 ± 1.93^a^
Sodium (meq/L)	154.83 ± 12.70^a^	124.17 ± 9.47^b^	154.50 ± 16.74^a^	153.67 ± 16.36^a^
Potassium (mmol/L)	4.78 ± 0.46^a^	2.48 ± 0.22^b^	4.66 ± 0.32^a^	4.54 ± 0.30^a^

-Results are expressed as Mean ± SD, n = 8. -Different characters denote significance between groups. -The mean difference is significant at P < 0.05.

## 4. Discussion

The late stages of aging are characterized by a decline in the internal organs weight (Spencer, 1996) which was reported in the current study. This was attributed to aging-related reductions in the lean body mass, muscle mass and strength, bone mineral density, and skin thickness (Matsumoto, 2002). These significant declines were not ameliorated after the administration of Ta-65 or pomegranate. 

Aging also affects the testes, reduces the testosterone level along with sperm production and erectile functions. FSH binds to its receptors on the Sertoli cells to stimulatespermatogenesis, where LH stimulates testosterone production by Leydig cells to act on the Sertoli and peritubular cells of the seminiferous tubules and stimulate spermatogenesis (Babu et al., 2004). Testosterone regulates normal growth, development of male sex organs, and the maintenance of secondary sexual characteristics. Testosterone also improves sperm motility, morphology, and epididymis functions (Gray et al., 2005). 

The serum levels of FSH, LH, and testosterone declined due to normal aging resulting in significant decreases in sperm count and motility in association with abnormal sperms. Deleterious histological abnormalities in testes were also reported. By normal aging, a decline in the sensitivity of the hypothalamus to feedback regulators occurs resulting in a progressive loss of homeostasis and alteration in the hormone production (Dilman et al., 1979). The weakening in the cellular antioxidant milieu in male reproductive system associated with aging was reported to inhibit the sperm axonemal phosphorylation resulting in increasing the number of immotile sperms (Desai et al., 2010). Aging induced DNA damage and affects DNA repair pathway in pachytene spermatocytes (Paul et al., 2011).

Administration of Ta-65 or pomegranate to aged rats improved serum levels of FSH, LH, and testosterone resulting in increased sperm quality; sperm count and motility as well as normal sperm morphology. Ta-65 exerted a partial preservation of the normal structure of the seminiferous tubules and Leydig interstitial cells. In vitro treatment of cultured Leydig cells with Ta-65 increased the number of Leydig cells and elevated the production of testosterone in the culture media (Jiang et al., 2015). Pomegranate stimulates GnRH from hypothalamus to increase synthesis and secretion of LH and FSH from the anterior pituitary (Hong et al., 2008). Pomegranate also improved the histological structure of testes with normal spermatogenesis, well preserved Sertoli cells and well delineated tubular basement membrane. The interstitium between tubules and Leydig cells was also intact (Utomo et al., 2019).

The decline in the renal function is a well-known aspect of normal ageing (Kafetz, 1983), with decreased kidney function and glomerular filtration rate (GFR) (Denic et al., 2016). In the current study, normal aging caused significant elevations in serum creatinine and uric acid. This could be attributed to aging-induced decline in renal blood flow and GFR as well as sclerosis of glomeruli, and inflammation or fibrosis (Zhou et al., 2008). 

Treatment of aged rats either with Ta-65 or pomegranate normalized the serum levels of creatinine and uric acid in accordance with previous studies (Kim et al., 2014; Cai et al., 2018). Ta-65 was shown to protect telomeres from shorting-induced renal tubules injury (Yu et al., 2018), reduced tubular proliferation, and reduced tubular autophagy (Westhoff et al., 2010). Oral administration of pomegranate ameliorated CCl4-induced nephrotoxicity via increasing the antioxidant defenses (Abdel Moneim and El-Khadragy, 2013). 

Normal aging causes imbalance in the levels of sodium and potassium (Prabhunath et al., 2016). Kidney is the central organ for maintaining the body’s sodium balance by controlling sodium reabsorption along different nephron segments. Sodium reabsorption is coupled to potassium secretion via the renal outer medullary potassium channels (Van der Wijst et al., 2018). Sodium and potassium are essential in maintaining cellular homeostasis, osmotic pressure and water distribution throughout the body, proper pH, regulating heart and muscles functions, oxidation-reduction reactions, catalysis as cofactors for enzymes, renal acid-base balance, and the conduction of nerve impulses. Therefore, abnormal levels of sodium and potassium lead to a variety of pathological disorders (Barrett et al., 2010).

The current study revealed significant declines in serum sodium and potassium levels due to aging. It was attributed to aging-induced decline in sodium reabsorption, GFR, tubular responsiveness to vasopressin, and renin activity. The decrease in potassium level was attributed to increased potassium translocation into cells and increased its loss into urine (Bardak et al., 2017). On the other hand, treatment of aged rats with Ta-65 or pomegranate normalized serum sodium and potassium levels.Ta-65 improved serum electrolytes in patients with chronic kidney diseases (Zhang et al., 2014). Pomegranate normalized the electrolyte levels disturbed by nicotine (Aboulgasem and Azab, 2014). 

## 5. Conclusions

Ta-65 and pomegranate supplements have antiaging activities against aging-induced male infertility and kidney dysfunction. Ta-65 and pomegranate supplements delayed normal aging by recovering male infertility in the form of good sperm analysis, sex hormonal balance, and normal testicular histology. In addition, Ta-65 and pomegranate supplements improved kidney function tests by normalizing serum levels of creatinine, uric acid, sodium, and potassium. Therefore, Ta-65 and pomegranate supplements are promising therapeutic agents for delaying the deteriorations induced by normal aging. Further studies are required to disclose the molecular mechanisms through which these agents act.

## Acknowledgment

Authors gratefully acknowledge Dr. Sayed Abdel Raheem, Assistant Professor of Histopathology, Faculty of Medicine for Boys, Al–Azhar University, Cairo, Egypt, for the assistance with the histological study.

## Disclaimers

Research Ethics Committee (REC) for animal research at National Hepatology and Tropical Medicine Research Institute (NHTMRI), Cairo, Egypt has approved the research protocol (Serial#18-2018). 
